# A Study on the Effect of Graphene Oxide on Geotechnical Properties of Soil

**DOI:** 10.3390/ma17246199

**Published:** 2024-12-18

**Authors:** Kyungwon Park, Ju-Hoon Kim, Junwoo Shin, Hoyoung Lee, Boo Hyun Nam

**Affiliations:** 1 Department of Civil Engineering, College of Engineering, Kyung Hee University, Yongin 17104, Republic of Koreakimjuhoon@khu.ac.kr (J.-H.K.);; 2 Department of Civil Engineering, Kumoh National Institute of Technology, Gumi 39177, Republic of Korea

**Keywords:** edge-oxidized graphene oxide (EOGO), direct shear strength, compressibility, contact angle

## Abstract

Edge-oxidized graphene oxide (EOGO) is a nano-sized material that is chemically stable and easily mixed with water due to its hydrophilic properties; thus, it has been used in various engineering fields, particularly for the reinforcement of building and construction materials. In this study, the effect of EOGO in soil reinforcement was investigated. When mixed with soil, it affects the mechanical properties of the soil–GO mixture. Various amounts of the GO (0%, 0.02%, 0.06%, 0.1%) were added into the sand–clay mixture, and their geotechnical properties were evaluated via multiple laboratories testing methods, including a standard Proctor test, direct shear test, compressibility test, and contact angle measurement. The experimental results show that with the addition of EOGO in soil of up to 0.06% EOGO, the compressibility decreases, the shear strength increases, and the maximum dry density (after compaction) increases.

## 1. Introduction

Graphene oxide (GO), consisting of a single atomic layer of carbon and modified with oxygen-containing functional groups, is typically synthesized through the chemical oxidation of graphite [1]. Increasing the reactivity of GO by functionalization allows it to serve as a seeding agent for hydration products and to promote the rapid development of calcium–silicate–hydrate (C-S-H) gel inside cementitious matrices [2,3,4,5,6]. With these properties, GO has been positioned a multifaceted additive in the construction area, where it serves as an enhancement of mechanical and structural properties [7,8,9,10,11].

**Figure 1:**
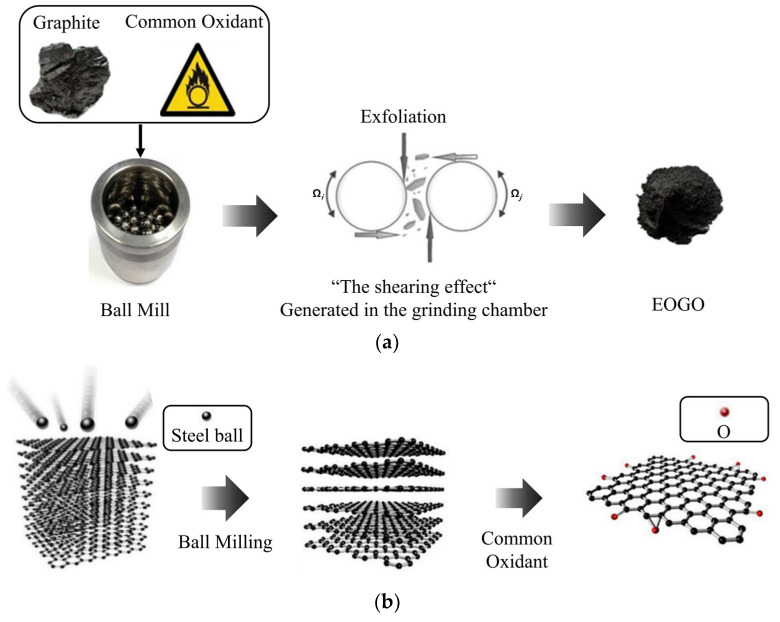
Schematic diagram of the EOGO manufacturing process [26]; (**a**) ball-milling and oxidant (**b**) mechanochemical processes of EOGO production.

Edge-oxidized graphene oxide (EOGO), examined in this study, represents an economical derivative of graphene oxide (GO). Using the ball-milling technique, EOGO is produced as multi-layered graphene flakes with reactive oxygen groups mainly located at the edges, as depicted in Figure 1. Unlike conventionally produced GO, EOGO features a lower concentration of reactive oxygen groups, enhancing its chemical stability and reducing the propensity for aggregation. This difference in oxygen group distribution enhances the interfacial interactions with cementitious matrices, leading to improved mechanical properties such as increased tensile strength and crack resistance compared to traditional GO. Furthermore, the reduced oxygen content contributes to a more efficient production process by lowering energy consumption and reducing the generation of hazardous by-products [12]. In addition, this manufacturing process significantly decreases costs by removing hazardous waste and disposal obligations [13,14,15,16]. This approach establishes EOGO as a potential alternative for enhancing cementitious composites, offering both performance improvement and cost feasibility, especially in extensive construction projects [17,18,19,20,21].

Edge-oxidized graphene oxide (EOGO) is comprehensively studied for its beneficial effects on cement composites, markedly improving rheological characteristics and mechanical strength, as assessed by recent investigations. Several studies demonstrate that EOGO can enhance the rheological properties and performance of fiber-reinforced concrete, resulting in improved durability and structural integrity [22,23,24]. The data indicate that EOGO’s interaction with cementitious materials enhances material characteristics at optimal concentrations of approximately 0.05%.

Although the advantages of EOGO in improving the compressive and flexural characteristics of cement pastes and mortar mixtures have been recorded [25,26,27,28,29,30,31], its interactions with soil have still been inadequately investigated. The capacity of the robust interactions with soil components are suggested by the enhanced GO surface with hydrophilic functional groups. These interactions may impact critical engineering properties, such as mechanical behavior, charge distribution, water retention, and oxygen availability, that directly influence soil stability and microbial activity [32,33,34,35,36,37]. However, there are limited studies assessing the effects of EOGO on soil properties [6,38].

In this study, a series of laboratory tests were performed to mechanically characterize the effect of EOGO in soils. The laboratory testing program includes compaction test, direct shear test, compressibility test, and water droplet contact angle measurement. The results of each test are presented, and a discussion on how EOGO affects the mechanical properties of soils is given below.

## 2. Materials

### 2.1. Soil and EOGO

**Figure 2:**
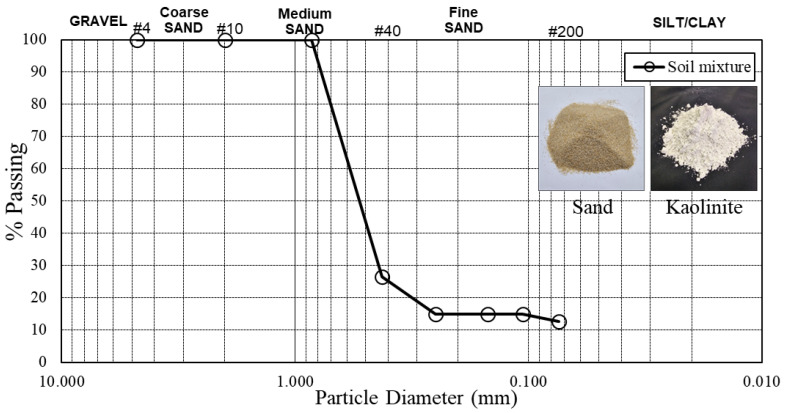
Particle distribution of the soil mixture (sand and clay).

The soil samples used in this study were a mixture of sand (Jumunjin standard sand), clay (calcined kaolin), and edge-oxidized graphene oxide (EOGO). The clay was calcined kaolin, which was made by calcining kaolin ore at a temperature of 1200 °C to increase purity and whiteness. The prepared soil mixture exhibited a maximum dry unit weight of 18.35 kN/m^3^ and an optimum moisture content of 13%, classified as SC (sandy clay) according to the USCS (Unified Soil Classification System) (Table 1). Figure 2 shows the particle distribution curve and a photograph of the soil mixture, providing a visual overview of its composition.

The soil composition utilized in this study comprised 85% sand and 15% clay, categorized as Clay Sand (SC) in the Unified Soil Classification System (USCS). The mean particle sizes of sand, clay, and edge-oxidized graphene oxide (EOGO) are 0.3–0.6 mm, 0.045 mm, and 450 nm, respectively. The physical characteristics of the soil mixture, encompassing maximum dry unit weight and optimum moisture content, are delineated in Table 1. Figure 2 illustrates the particle distribution of the soil mixture. 

**Figure 3:**
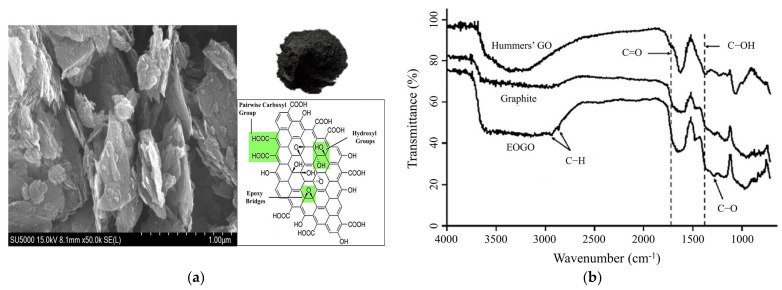
Material characterization of the EOGO: SEM (**a**) and FTIR (**b**).

Figure 3a provides scanning electron microscopy (SEM) pictures of EOGO, while Figure 3b shows the associated results from molecular infrared spectroscopy (FTIR analysis), illustrating a comprehensive characterization of the material’s microstructure and chemical properties. The physical features of EOGO are summarized in Table 2. EOGO has a specific gravity of 1.91, signifying that it is denser than water, thus affecting its distribution within the soil matrix. The carbon content of 90–95% indicates a substantial presence of graphene material, enhancing its strength and structural integrity. The oxygen concentration of 5–10% is due to the oxygenated functional groups on the margins of the nanosheets, which augment the hydrophilic properties of EOGO and facilitate improved contact with soil particles and water. With a surface area of 200–300 m^2^/g, EOGO exhibits high reactivity, which facilitates strong bonding within the soil matrix, improving soil stability. A mean particle size of 450 nm and thickness of approximately 10 nm suggests that EOGO consists of nanosheets capable of filling voids between soil particles, which significantly alters the mechanical properties of the soil. Furthermore, EOGO’s density of 1.0 g/cm^3^ allows for efficient dispersion in water, ensuring uniform mixing with the soil.

### 2.2. Specimen Preparation

**Figure 4:**
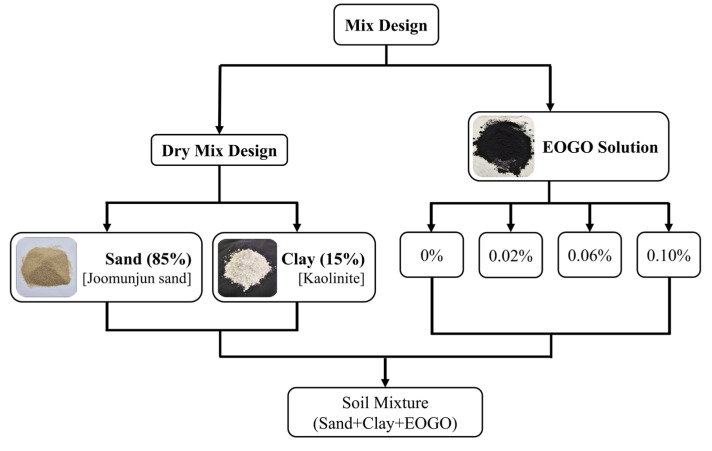
Experimental design.

In this study, four types of mixtures were designed by increasing the GO content in soil (see Figure 4). To mix the sand and clay, a dry mixing method (mechanical shearing) was employed for 3 min at a ratio of 80% sand to 15% clay of the total soil sample weight. Subsequently, the amount of EOGO was added to the soil mixture at weights of 0%, 0.02%, 0.06%, and 0.1%. The experimental design is illustrated in Figure 3.

When mixing the EOGO with soil samples, a wet mixing method was selected to transform the materials into an aqueous solution. Based on the authors’ previous experiences, the dry mixing method results in an issue where the EOGO is not evenly spread over the sample. For the better dispersion of EOGO in the soil mixture, a wet mixing method was used. During wet mixing, the concentration of the aqueous solution was mixed at the ratio of the amount of EOGO required per 30 g of water. The solution was prepared over 3 h using a high-speed stirrer SSC611CA model from Matsushita (now Panasonic) at a speed of 2000 rpm. The mixture was stirred and mixed for 3 min. This specific duration and speed were chosen based on preliminary trials that indicated that these conditions were optimal for achieving a homogeneous mixture of EOGO within the soil. The particle size of the experimental soil sample is 0.3~0.6 mm for sand and 0.045 mm for clay, which is much larger than EOGO, so the EOGO aqueous solution can be easily dispersed. The exact fraction of EOGO aqueous solution was prepared according to the mixing ratio shown in Table 3.

## 3. Testing Methods

### 3.1. Direct Shear Test

**Figure 5:**
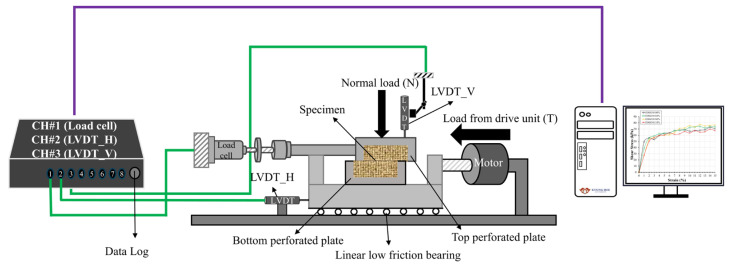
Schematic diagram of the direct shear test.

For the direct shear test, we prepared the specimens with the same relative density, and a horizontal force (shear force) was applied in the horizontal direction while applying normal stress. Figure 5 is a side view of the direct shear testing device used in this study. The model is a DA-525 model from Dong-A Testing Machinery, a digital direct shear tester. The direct shear tester performs horizontal shearing at a constant speed control of 1 mm/min. The dimension of the shear box is 60 mm in width and 20 mm in height.

Three vertical stresses of 45, 90, and 130 kPa were applied per sample in accordance with ASTM 3080 [41], and the shear rate during the direct shear test was set with 1 mm/min. The specimen used in the direct shear test was a mixture of mixed soil samples and various concentrations of the EOGO aqueous solution (0%, 0.02%, 0.06%, and 0.1%), and the relative compaction level was set to 90% in the shear box. The moisture content of the specimen was kept as 15%.

### 3.2. Compressibility Test

The compressibility test was conducted using an oedometer device. The purpose of this experiment was to evaluate the effect of EOGO on soil’s compressibility. The maximum shear strength was identified when 0.06% of EOGO was added; thus, the test was conducted on the specimen with 0% (control) and 0.06% EOGO. For each amount of EOGO content, three specimens were tested, and the results were averaged. All samples used in the experiment were saturated for more than a day, and the vertical stress was applied in six stages at 250, 500, 750, 1000, 1250, and 1500 (kPa), using the weight used for normal consolidation. To determine the duration of each load, the specimen was loaded for 24 h, and then the time at which no further settlement occurs was determined for the next-loading step. The test results showed that the level of settlement was constant after 4 h, so the load was applied based on 6 h.

### 3.3. Compaction Test

The standard Proctor test was conducted to evaluate the compaction characteristics of the GO–soil mixture. This test identifies the maximum dry unit weight and optimal water content ratio of the sample depending on the presence or absence of EOGO. A compaction test was conducted in accordance with ASTM D698 [42]. Compaction was performed by hammering the sample 25 times in three layers. The compaction test was performed on samples with 0% and 0.06% EOGO content. The maximum unit weight (*γ*_dmax_) was also used to determine the target relative compaction when preparing the specimen for the direct shear test.

### 3.4. Contact Angle Measurement

A water droplet contact angle measurement was employed to characterize the basic surface properties of the EOGO. The surface properties of a material are closely related to the chemical properties that make up the material. The contact angle test measures the angle between the flat surface of a solid and the liquid surface. It measures the angle between three thermodynamic parameters: liquid–gas surface tension (*γ_LG_*), solid–gas surface energy (*γ_SG_*), and solid–liquid interfacial tension (*γ_SL_*). Based on the relationship, it is defined through Equation (1) [43,44,45,46].

(1) $$\gamma_{SG}=\gamma_{LG}cos\theta +\gamma_{SL}$$

where *γ_SG_* represents the solid–gas surface energy, *γ_LG_* is the liquid–gas surface tension, *γ_SL_* denotes the solid–liquid interfacial tension, and *θ* is the contact angle formed between the droplet and the solid surface.

Because it is difficult to identify the contact point in the three phases, in this study, the contact angle was expressed as the apparent contact angle, and the hydrophobicity of EOGO was determined. The testing method followed ASTM D5946-09 [47].

**Figure 6:**
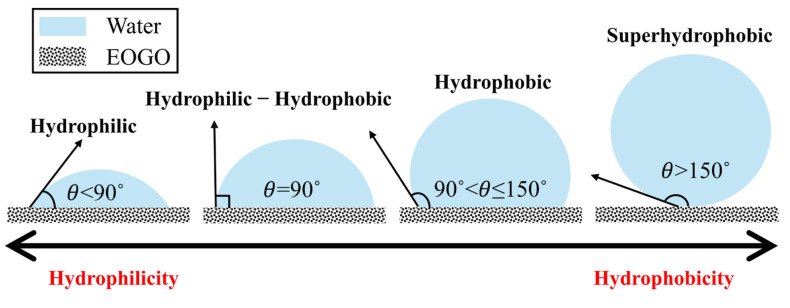
Classification of the contact angle measurement between EOGO and the water droplet.

Figure 6 shows the four classifications of the contact angle of the water droplet that represents the degrees of hydrophilic and hydrophobic. When the contact angle between a water droplet and the surface is small, it is defined as hydrophilic and wettable. On the other hand, when the contact angle is large, it is defined as hydrophobic [46]. The purpose of this test is to simply discern the hydrophilic and hydrophobic characteristics of the materials used in the test.

As a test method, the sample was produced by drying the EOGO sample at a temperature of 50 °C. Afterwards, the EOGO sample was placed on a flat glass plate, and the water droplet (50 μL) was gently placed on the sample surface. At this time, it should not be dropped or dispersed on the surface. Subsequently, the water droplets that formed on the surface were photographed. The contact angle measurement method was utilized to measure the apparent contact angle on both sides of the water droplet; the contact angle was calculated using Equation (2), and the average values of the angles were measured 10 times.

(2) $$\theta^{\prime }=\left(\frac{\alpha_{1}-\beta_{1}}{2}+\frac{\alpha_{2}-\beta_{2}}{2}+\frac{\alpha_{3}-\beta_{3}}{2}\right)/3$$

where θ′ denotes the apparent contact angle, and *α*_i_ and *β*_i_ (i = 1, 2, and 3) represent the measured apparent contact angles on both sides of the water droplet for the three measurements, respectively.

## 4. Results and Discussion

### 4.1. Shear Strength

**Figure 7:**
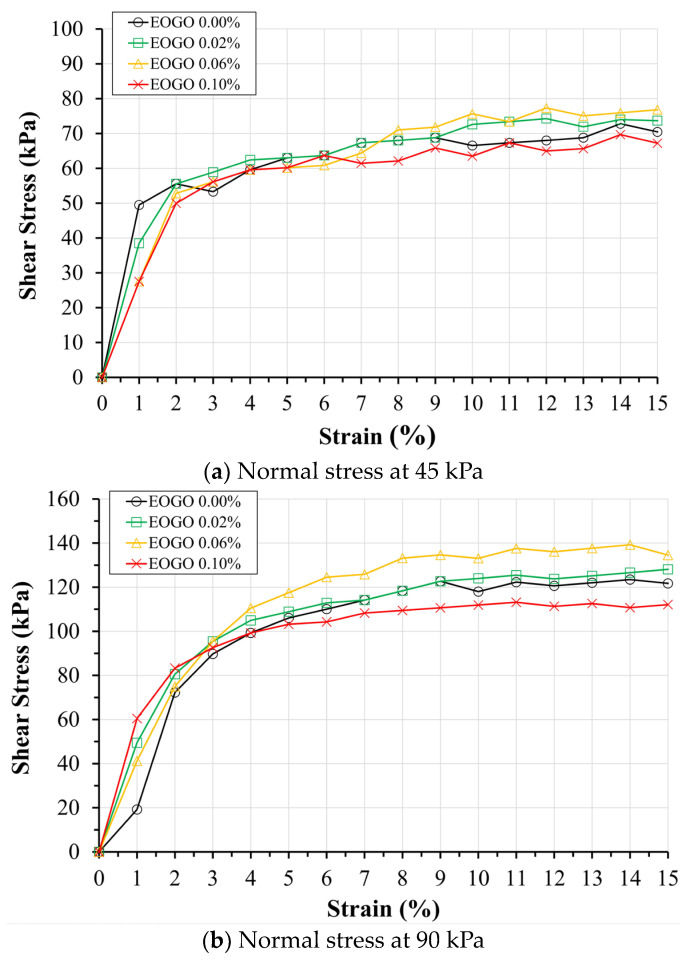

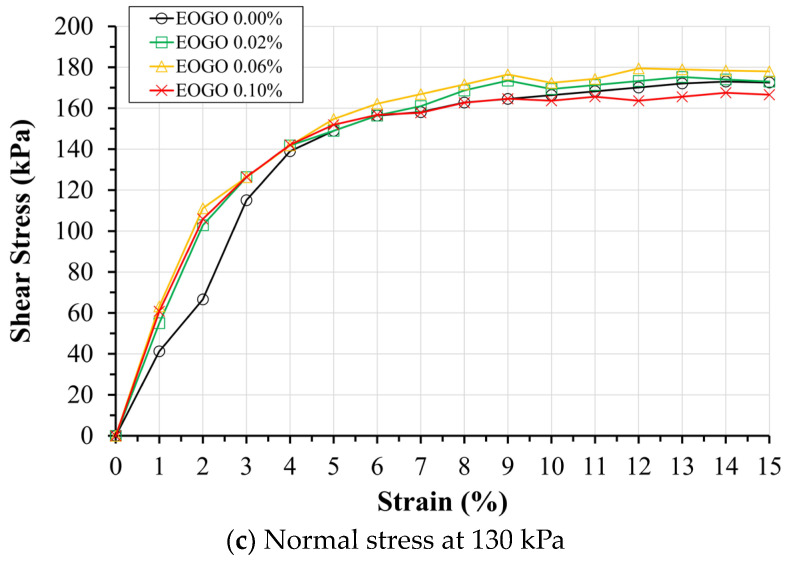
Results of the direct shear strength test.

Figure 7 shows the stress–strain curves of the direct shear testing on the soil–EOGO mixture. As the normal stress increases, the shear stress increases. Overall, the shear stress increases with increasing the EOGO content, exhibiting the largest shear stress at 0.06%. However, after 0.06% EOGO, the results show a decreasing trend at 0.1%. As the normal stress increases, adding EOGO causes an increase in the stiffness at lower strain levels. In particular, at normal stress levels of 90 kPa and 130 kPa, this trend is clearly shown. Notably, all stress–strain curves do not show clear transition points, which can be attributed to the characteristics of the Clay Sand (SC) used and to the testing methodology defined by ASTM D3080 [41]. According to ASTM D3080, failure in a shear test is defined as the condition at maximum shear stress or at a shear stress corresponding to 15 to 20 percent relative lateral displacement. In our experiments, we consistently conducted tests to a strain of 15%, aligning with this criterion.

**Figure 8:**
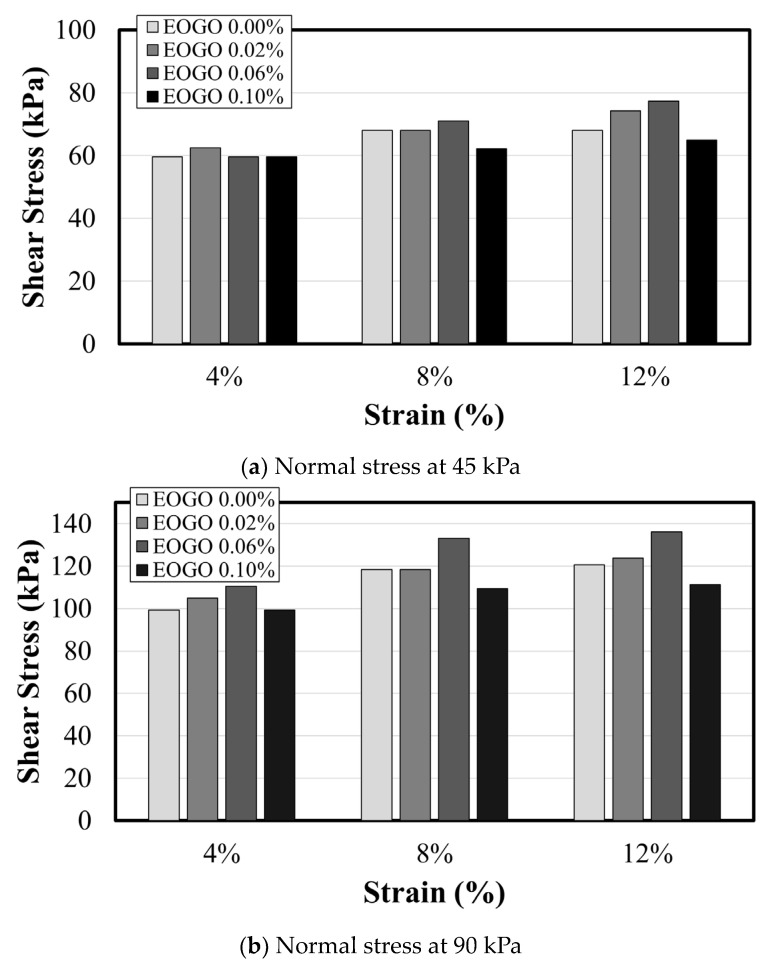

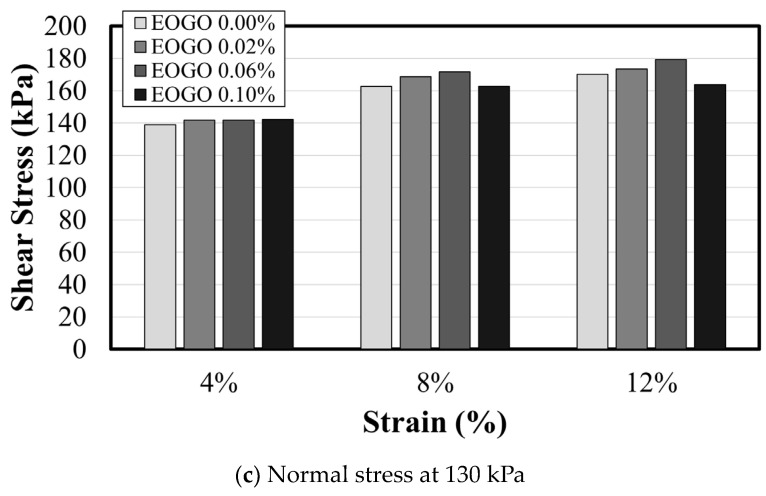
Comparison of the shear strength of the soil–EOGO mixture determined at various strain levels.

All stress–strain curves do not show clear transition points; thus, it is difficult to identify the maximum shear stress that corresponds to the strength. The use of ASTM D3080 demonstrates that failure is defined as the maximum shear stress or the shear stress at 15 to 20% relative lateral displacement. Following this standard, the test is conducted at a 15% relative lateral displacement. To analyze the characteristics of shear stresses at different strain levels, several points were selected to determine “shear strength”. A transition appears in the range from 2% to 6% of the shear strain; thus, 4% was selected as a criterion of the “yielding” point. The selected strain ranges were 4, 8, and 12%, which were set as the standard when the strain increased by 4%. Figure 8 shows the values of shear stress at the selected strain levels of 4, 8, and 12%.

**Figure 9:**
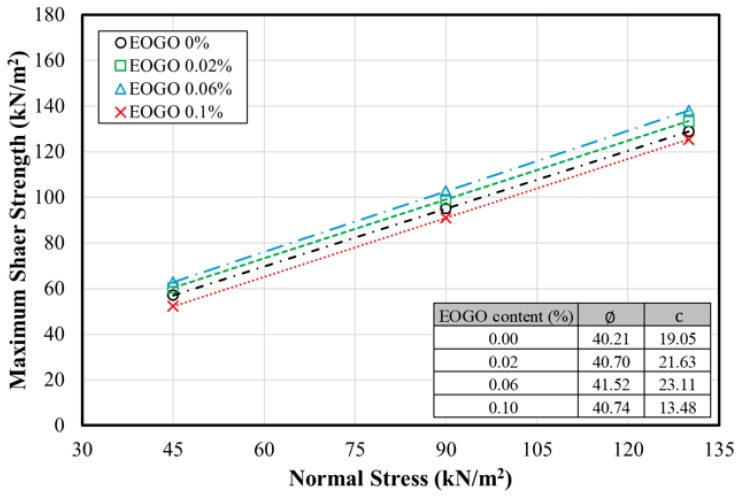
Shear strength of the soil–EOGO mixtures with varied EOGO content.

Based on the peak shear stresses selected at the strain of 8%, the shear strength of the soil–EOGO mixture were determined (see Figure 9). The cohesion increases with increasing EOGO content, exhibiting a maximum cohesion at 0.06% of EOGO; however, 0.1% EOGO clearly shows a drop in shear strength. The internal friction angle shows a similar trend but the change due to the EOGO content is not significant, with a range of friction angles of 40.21 to 41.52 degrees.

### 4.2. Compressibility

**Figure 10:**
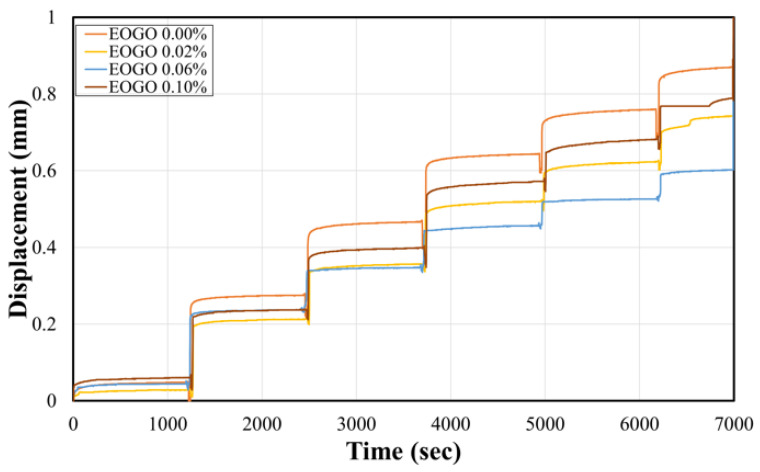
Result of the compressibility test (plot of displacement vs. time).

During the compressibility test, following the testing procedure of ASTM D2435 [48], the incremental loading level includes 250, 500, 750, 1000, 1250, and 1500 kPa. Under each load level, vertical deformation was continuously recorded over time until no further deformation occurred. Subsequently, the next level of loading was set, and the induced deformation was continuously recorded. Figure 10 shows the time series of displacement data for the soil–EOGO mixtures when incremental loads were applied. Although the initial displacement values under the first load level (250 kPa) were slightly different, the overall displacement profiles show distinguishable trends. EOGO 0% exhibited the largest displacement, which indicates the highest level of compressibility. On the other hand, EOGO 0.06% showed the lowest displacement, which indicates the lowest compressibility. 

**Figure 11:**
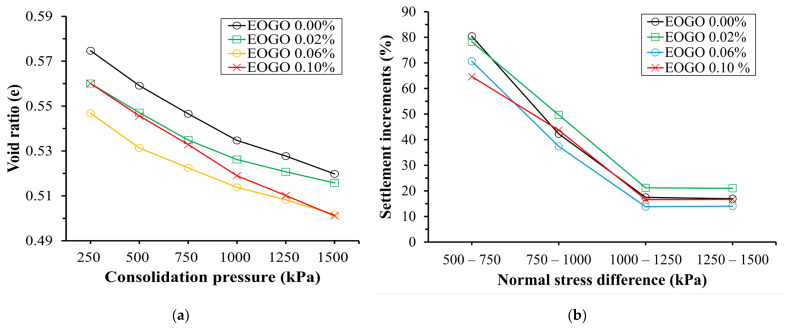
Results of the compressibility test: (**a**) void ratio vs. normal stress; (**b**) percentage of displacement increment vs. normal stress.

Based on the displacement measurement from the oedometer test, the void ratio (e) was computed at each loading level. As the consolidation load increases, the void ratio decreases. Figure 11a shows the void ratios under each load. Like the trend shown in Figure 9, the result shows that EOGO 0% shows the greatest void ratio while EOGO 0.06% exhibits the lowest void ratio under all loading levels. Additionally, the percentage of displacement increment was computed, and the results are presented in Figure 11b. It is clearly shown that the specimen with EOGO 0.06% has a settlement of 43.17% lower than the control specimen. It is believed that 0.06% EOGO is the optimum ratio that creates the highest density in the mixture. For the control (0% EOGO), the largest compressibility took place at the initial load increment (from 500 to 750 kPa) but showed lower compressibility as the loading level increases.

### 4.3. Compaction

**Figure 12:**
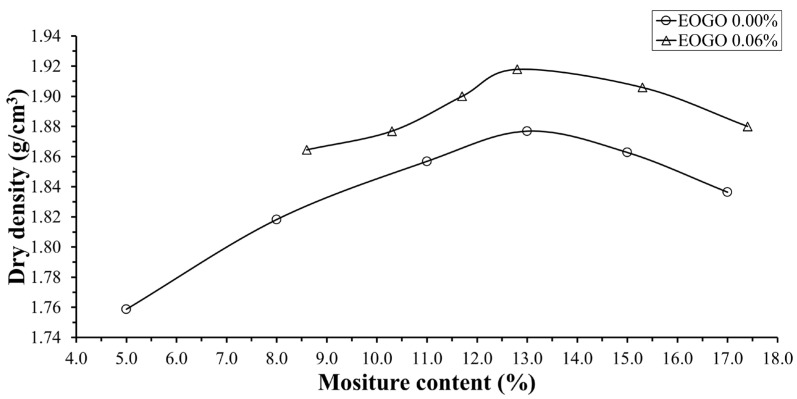
Result of the compaction test.

Figure 12 shows the result of the compaction test. In order to evaluate the effect of EOGO, two conditions were tested: 0% EOGO (control) and 0.06% EOGO. When EOGO was added into the soil mixture, the compaction curve was shifted upward and the maximum dry unit weight was increased from 1.85 g/cm^3^ to 1.92 g/cm^3^, while the optimal moisture ratio has a no significant change. Similar trends were reported in the previous studies using a soil–cement mixture [49,50,51,52]. EOGOs are spatially distributed throughout the soil mixture and cause a denser condition with the same level of compaction energy.

### 4.4. Result of Contact Angle Measurement

**Figure 13:**
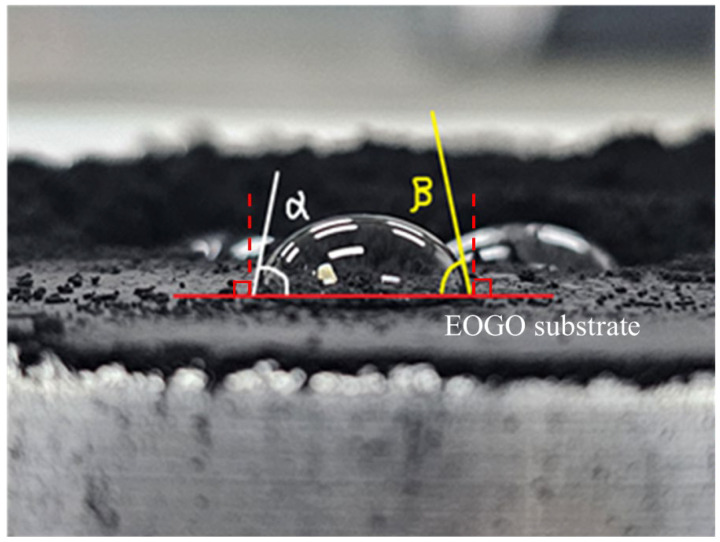
Result of the contact angle measurement.

It is believed that EOGOs are hydrophilic like typical GOs. In this study, considering the chemical properties of EOGO, their hydroxyl group content is lower than that of typical GOs, by 5–10%, but the degree of hydrophilicity is not known; thus, the contact angle measurement test was performed. Figure 13 is a photo of a water droplet placed on the surface of the EOGO. The apparent contact angle (θ) was computed using Equation (2). The apparent contact angle was measured 10 times, and the average contact angle was 88.3 degrees with a standard deviation of 0.675. This contact angle is lower than 90 degrees, which indicates hydrophilic characteristics but lower than typical levels of GOs.

## 5. Conclusions

In this study, the effects of EOGO on soil’s mechanical properties were investigated, particularly geotechnical properties of the soil–EOGO mixture. The conclusions derived from our research are given below.

EOGO is a nanomaterial, with a high specific surface area (200–300 m^2^/g) and hydrophilic attributes. Our findings indicate that the combination of EOGO and sand–clay soil resulted in good dispersion and the formation of hydrophilic nanosheets. These nanosheets fill the interparticle gaps (filling effect) and also affect soil–EOGO structure, resulting in a higher ratio of bound water to free water. This interaction led to the diminished compressibility of the soil samples. The infilling of voids with EOGO results in a denser soil matrix, reducing the likelihood of compression under applied loads and improving the overall stability of the soil. 

The examination of shear strength indicated that EOGO content of up to 0.06% enhanced soil’s shear strength parameters. The improvement is probably due to the efficient dispersion of EOGO particles, which enhanced interparticle bonding and offered mechanical reinforcement. The increased shear strength indicates that optimal EOGO concentrations can improve the structural performance of soil in engineering applications, such as foundations and embankments, where mechanical stability is essential. On the other hand, beyond the 0.06% EOGO content, the shear strength decreases. This decrease is likely due to the aggregation of EOGO nanosheets at elevated concentrations, which disturbs the uniform distribution and forms weak zones within the soil matrix.

For the compaction test, the addition of EOGO at an optimum content of 0.06% increases the maximum dry unit weight from 1.85 g/cm^3^ to 1.92 g/cm^3^. The optimum water content did not change with the addition of EOGO.

Further investigations could explore the long-term stability and ecological impact of EOGO-enhanced soils under various environmental conditions to assess durability and sustainability. Additional studies could also examine the effects of varying EOGO concentrations and their interactions with different soil types in order to optimize its use in broader geotechnical applications. Field trials could validate laboratory findings and facilitate the development of practical guidelines for EOGO implementation in soil stabilization projects, ensuring environmental safety and technical efficacy. 

## Figures and Tables

**Table 1:** Properties of the soil mixture.

Property	Value	ASTM Standards
Max. dry unit weight (kN/m^3^)	18.35	ASTM D1557-12e1 (2012) [39]
Optimum moisture content (%)	13	
USCS	Clay Sand (SC)	ASTM D2487-17 (2017) [40]

**Table 2:** Properties of the EOGO.

Property	Value
Specific gravity	1.91
Carbon (%)	90–95
Oxygen (%)	5–10
Surface area (m^2^/g)	200–300
Mean particle size (nm)	450
Thickness (nm)	~10
Density (g/cm^3^)	1.0

**Table 3:** Mix proportions of the specimens (GO–soil mixture).

Mix ID	Mix Identification	Sand (g)	Clay (g)	EOGO Solution (mg/mL) (EOGO + Water)
SCGO00	GO 0%	212.5	37.5	0
SCGO02	GO 0.02%			1.33
SCGO06	GO 0.06%			4
SCGO10	GO 0.1%			6.67

## Data Availability

The original contributions presented in this study are included in the article. Further inquiries can be directed to the corresponding author.
